# Microfluidic nano-plasmonic imaging platform for purification- and label-free single small extracellular vesicle characterization

**DOI:** 10.1038/s44328-025-00047-w

**Published:** 2025-07-30

**Authors:** Omid Mohsen Daraei, Avinash Kumar Singh, Saswat Mohapatra, Mohammad Sadman Mallick, Abhay Kotnala, Wei-Chuan Shih

**Affiliations:** 1 Department of Electrical and Computer Engineering, University of Houston, 4800 Calhoun Road, Houston, TX 77204 USA; 2 Department of Biomedical Engineering, University of Houston, 4800 Calhoun Road, Houston, TX 77204 USA; 3 Department of Chemistry, University of Houston, 4800 Calhoun Road, Houston, TX 77204 USA; 4 Program of Materials Science and Engineering, University of Houston, 4800 Calhoun Road, Houston, TX 77204 USA

**Keywords:** Imaging and sensing, Optics and photonics, Nanobiotechnology

## Abstract

Tumor-derived circulating small extracellular vesicles (sEVs) are a promising class of non-invasive biomarkers for disease diagnosis. However, their quantitative detection remains challenging due to their small size and the complexity of blood plasma. Therefore, sample preparation, such as purification and fluorescence labeling, is required. This study presents a purification-free approach using a microfluidic chip integrated with PlAsmonic NanO-apeRture lAbel-free iMAging (PANORAMA) for label-free single sEV characterization in plasma. CD63, CD9, and CD81 antibodies, specific for most sEVs surface antigens, are functionalized on arrayed gold nanodisks on invisible substrates (AGNIS) for selective capture. The automated microfluidic platform minimizes operational errors and biases and enables precise control of flow rates, directions, media volume, and composition for optimization. This platform requires only 20 µL of plasma, and the analysis is completed within 60 minutes. This platform shows great potential as a sensitive and effective tool for detecting and characterizing circulating sEVs without purification or labeling.

## Introduction

Small extracellular vesicles (sEVs), such as exosomes, play a role in intercellular communication locally and non-locally via blood circulation^1–4^. These vesicles are found not only in blood but also in nearly all other biological fluids. Originating from the endosomal pathway, sEVs range from 30 to 150 nm in diameter, with an average size of approximately 100 nm^5^. The surface proteins and cargo contained within sEVs are associated to their cellular origin, making them a valuable subject of study for understanding biological processes^6,7^. Precise identification and characterization of sEVs are essential for exploring their role in normal physiological processes and disease-associated mechanisms^8,9^. Conventional sEV characterization methods, such as western blot, enzyme-linked immunosorbent assay, and bead-based flow cytometry, have significant limitations in assessing the heterogeneity of individual sEV^10–16^. Additionally, these methods require relatively large volumes of biofluid samples for each assay and are not well-suited for smaller sample inputs^17–20^. More importantly, these techniques typically require prior isolation and purification, which are time-consuming, yield low recovery rates, and lacks reproducibility, thus making their translation toward clinical applications challenging^21–23^.

Microfluidic technologies offer compact, efficient, and integrated solutions for analyzing sEVs by overcoming challenges associated with traditional fluid-handling. These platforms enable precise manipulation of small sample volumes and automated sample handling with high reproducibility^24^. Recent innovations in microfluidic systems combined with optical detection have further advanced sEV analysis. For example, Zhao et al. developed the ExoSearch chip, a microfluidic platform that utilizes immunomagnetic beads for the enrichment and detection of blood plasma exosomes^25^. Xu et al. developed a phosphatidylserine-based capture and detection chip that integrates magnetic isolation with fluorescence readout via a label-free DNA aptasensor for efficient exosome interrogation^26^. Wu et al. introduced a microfluidic-assisted enrichment strategy based on metabolic glycan labeling and click chemistry to selectively capture and fluorescently detect nascent EVs, enabling time-resolved analysis of their response to immunotherapy^27^. Moreover, to improve tumor-specific EV isolation, Reátegui et al. developed the EV herringbone chip, a microfluidic platform that leverages engineered nanostructures to enable rapid EV isolation with 94% specificity and a detection limit of 100 EVs/mL using fluorescent labeling strategies within 3 h^28^. Despite the wide use of fluorescence-based techniques for sEV analysis, these approaches often require multiple labeling and washing steps involving fluorescent dyes, quantum dots, or gold nanoparticles, thereby increasing assay complexity and cost^29,30^. As a result, the need for alternative, label-free detection methods have increasingly been recognized as they offer reduced operational complexity, preserve native biological structures, avoid potential interference from dyes or tags, and enable real-time, quantitative analysis without any effect of photobleaching.

Plasmonic sensing has emerged as a promising label-free alternative by enabling the real-time analysis of sEVs in their native state^31–33^. For instance, Im et al. demonstrated a plasmonic chip based on periodic nanohole arrays for exosome detection and surface protein characterization by monitoring spectral shifts in transmitted light resulting from refractive index changes upon exosome binding^8^. Chen et al. introduced a microfluidic device employing localized surface plasmon resonance (LSPR) for real-time, label-free detection of exosomes^34^. Our group has recently developed an imaging technique called PlAsmonic NanO-apeRture lAbel-free iMAging (PANORAMA), designed for label-free detection of nanoparticles^30^. This technique leverages the LSPR of arrayed gold nanodisks on invisible substrates (AGNIS) to detect nanoparticles as small as 25 nm^30^. AGNIS features high-density gold nanodisks fabricated on a glass substrate with an undercut structure, enhancing local electric fields and generating a blue-shifted LSPR due to radiative coupling^35,36^. The AGNIS platform facilitates single-vesicle detection using visible light in complex biological environments.

**Fig. 1:**
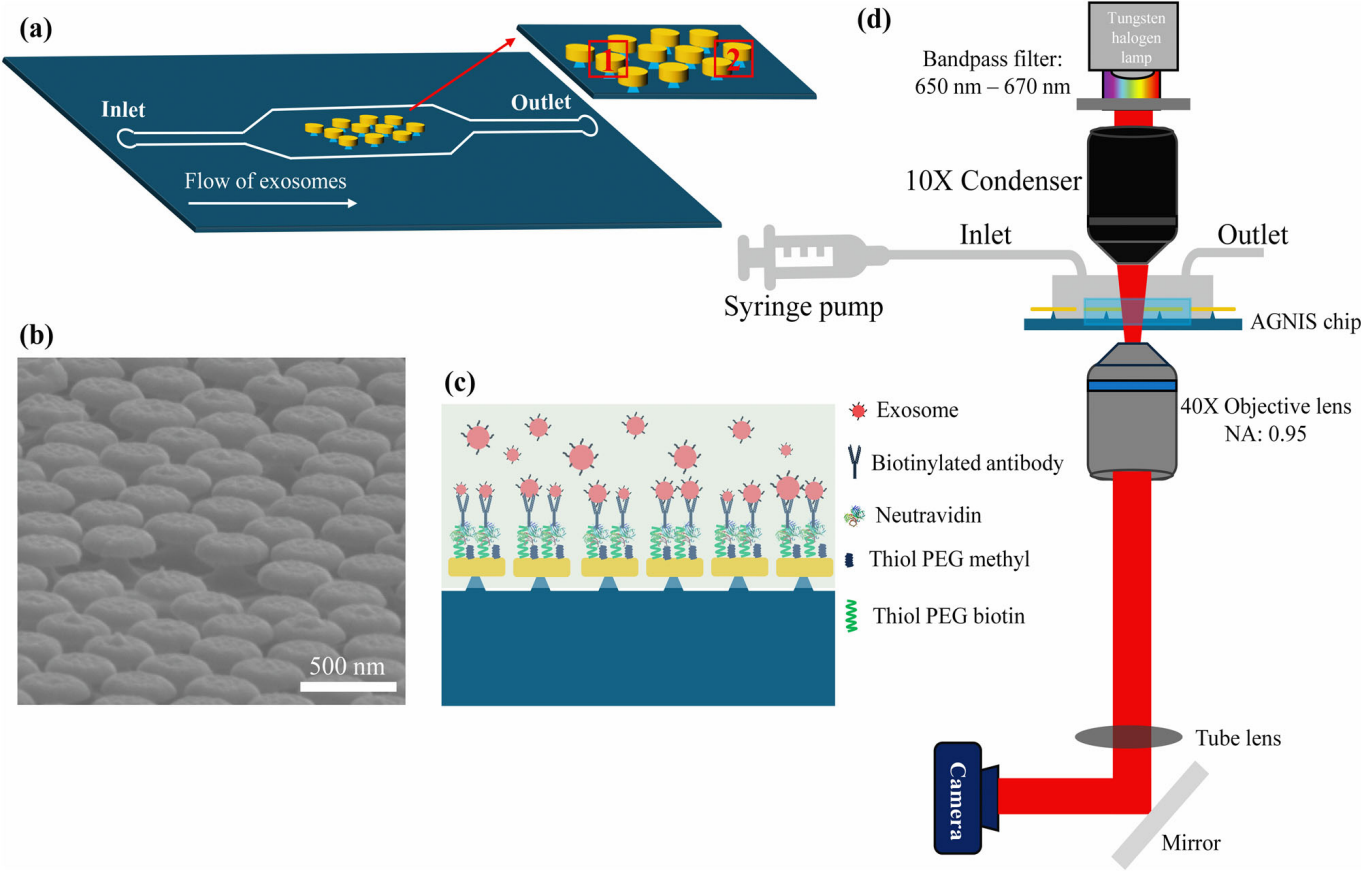
Overview of microfluidic nano-plasmonic imaging platform. **a** Conceptual illustration of the microfluidic device. **b** Scanning electron microscope (SEM) image of the AGNIS surface. **c** Schematic of AGNIS surface functionalization for exosome capture from human plasma. **d** Schematic of the optical setup for PANORAMA with the microfluidic device at the sample position.

In this study, we integrate PANORAMA with a custom-designed microfluidic platform to enhance assay automation, reduce sample volume requirements, and improve detection reproducibility, as shown in Fig. 1a. The device utilizes a push-pull flow strategy within the microchannel to optimize sEV capture efficiency and improve detection sensitivity. The microfluidic system offers precise control over flow rate, direction, and media volume and composition, supporting optimal assay conditions. The integrated microfluidic-PANORAMA platform automates the entire sEV capture and detection process pneumatically, enabling high-throughput processing and increased detection accuracy. We demonstrate the platform’s capability by directly detecting and quantifying plasma-derived exosomes within the microfluidic system. Notably, the system enables direct analysis of unprocessed plasma samples, requiring only 20 µL of plasma from liver cirrhosis patients to quantify circulating sEVs. Overall, this integrated approach offers a robust and scalable solution for real-time sEV analysis and supports a wide range of applications in exosome-related biomedical research and clinical diagnostics.

## Results

### LSPR measurement of AGNIS and the working principle of PANORAMA

PANORAMA leverages the high refractive index sensitivity of AGNIS’s LSPR to detect nanoparticles. AGNIS shows high refractive index sensitivity due to the radiative coupling of gold nanodisks and an undercut structure to expose the region with the highest plasmonic electric-field enhancement^35–38^. We first measured the LSPR spectrum of the fabricated AGNIS patches and determined their refractive index sensitivity using a custom-built optical setup (see Supplementary Note 1 for details). AGNIS exhibited LSPR peak wavelengths of 659 nm in air and 739 nm in water, yielding a sensitivity of 242.42 nm/RIU, as shown in Supplementary Fig. 1. This high sensitivity has proven effective in detecting nanoparticles via PANORAMA in previous studies^30,37,39^. As the target approaches the AGNIS, the local refractive index increases, causing a redshift in the LSPR extinction curve. This redshift decreases extinction and enhances localized light transmission within the operating narrowband wavelength, creating a virtual nanoaperture beneath the target. The enhanced transmission appears as an increasing pixel brightness at the particle’s position on AGNIS. To maximize the signal-to-noise ratio, PANORAMA operates by illuminating with filtered light centered near the steepest portion of the LSPR spectrum’s left tail shoulder. This enables PANORAMA to detect sub-100 nm nanoparticles, including weakly scattering biological particles, with high sensitivity and precision.

### Purified exosome detection using an integrated microfluidic-PANORAMA platform

To demonstrate the detection capabilities of our AGNIS-integrated microfluidic device, we first used it to detect purified exosomes extracted from the serum of a liver cirrhosis patient by a published protocol^40^. The AGNIS surface was functionalized with exosome-specific antibodies, as described in the “Methods” section, to facilitate exosome capture. A $\sim \,$200 µm × 200 µm area of AGNIS, labeled as region 1 in Fig. 1a, was selected as the sensing area within the channel. The channel was initially filled with PBS-1X, and background optical images of AGNIS were acquired. Subsequently, 20 µL of the purified exosome in PBS-1X with a concentration of 7.2 × 10^5^ exosomes/µL was delivered into the microfluidic channel. To first fill up the channel, the flow continued for 1 min at a flow rate of 5 µL/min. The total channel volume, including the inlet and outlet port, was ~5.25 µL. As the channel is filled, exosomes start binding to the AGNIS surface. To enhance binding, a “push-pull” incubation strategy was implemented using a pre-programmed syringe pump. In this approach, the exosome solution was alternately flown forward and backward within the channel at a flow rate of 1.5 µL/min, with each “push” and “pull” cycle lasting for 10 min. In other words, 15 µL forward flow passed by the AGNIS sensing region during the “push”, and the same amount flew backward during the “pull”. This approach allows the entirety of the sample to be cycled through the microfluidic channel. It also resolves the issue of depleting a small amount of fluids prior to sufficient exosome-AGNIS interactions with unidirectional flow. The push-pull flow was paused temporarily during image acquisition to reduce motion artifacts.

**Fig. 2:**
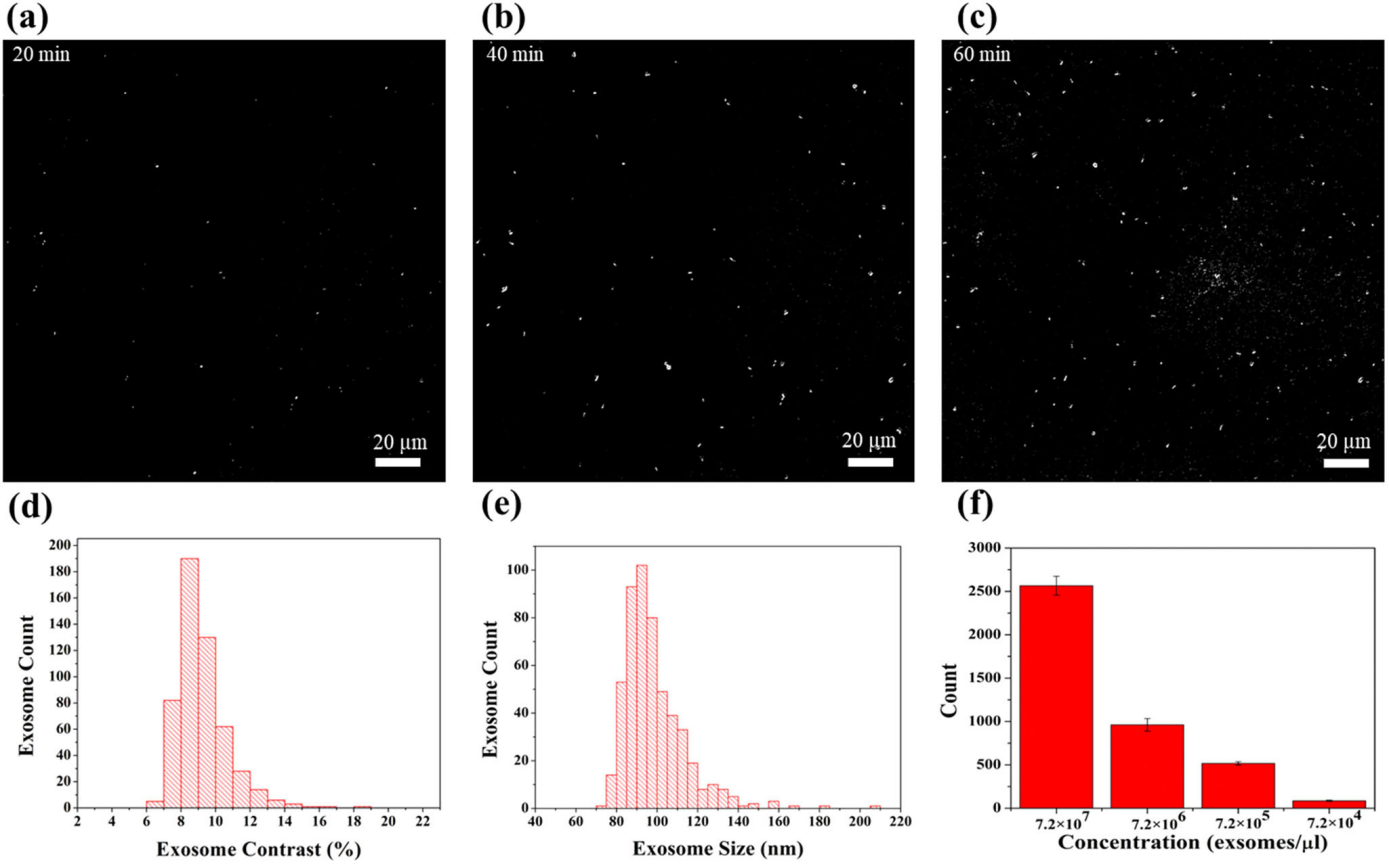
Detection of purified exosomes using PANORAMA on the microfluidic device. **a**–**c** Time-lapse PANORAMA images showing purified exosome detection over 60 min, with images captured every 20 min. **d** Contrast histogram of detected exosomes. **e** Size histogram of the detected exosomes. **f** Column bar plot representing the average counts and standard deviations for detected exosomes at four different concentrations, and each concentration was measured in three independent experiments.

Optical images were taken every 20 min after the purified exosome solution was introduced. The entire process, including sample injection, incubation, and image acquisition, was automated, requiring no manual intervention. Figure 2a–c shows time-lapse PANORAMA images of detected exosomes over 60 min. After 20 min of “push-pull” incubation, 73 particles were detected, and steadily increased to 537 by the end of 60 min (Fig. 2c). This illustrates time-lapsed accumulative capturing of exosomes via antibody-antigen binding. Notably, background noise remained constant at approximately 1% throughout the experiment, demonstrating the microfluidic device’s robustness against external influences compared to open systems. The contrast of exosomes extracted from the PANORAMA images was 9.1 ± 1.5%, as shown in Fig. 2d. Notably, the PANORAMA contrast is directly correlated with particle size. By comparing PANORAMA contrast with the size distribution obtained from nanoparticle tracking analysis (NTA), a relationship was established previously, *Y* = 0.153*X*^2^ + 7.096*X* + 20.211, where *X* represents PANORAMA contrast (%) and *Y* denotes exosome diameter in nanometers^37^. Figure 2e shows that the mean size of the detected exosomes was 97.45 ± 15 nm obtained from the PANORAMA image (Fig. 2c).

Figure 2f shows the average exosome counts and the standard deviations for four serially diluted exosome concentrations, ranging from 7.2 × 10^7^ to 7.2 × 10^4^ exosomes/µL. A concentration-dependent trend is observed: higher sample concentrations yielded greater numbers of detected exosomes. At the highest concentration (7.2 × 10^7^ exosomes/µL), the average count was 2565 ± 109, while the lowest concentration (7.2 × 10^4^ exosomes/µL) yielded a mean of 85 ± 7. Intermediate concentrations of 7.2 × 10^5^ and 7.2 × 10^6^ exosomes/µL resulted in average counts of 516 ± 19 and 962 ± 72, respectively. Each concentration level was assessed in triplicate, with consistent particle counts and contrast measurements observed across all trials, indicating high reproducibility. Detailed datasets, including individual count and contrast values, are available in Supplementary Note 2.

### Single sEV detection from patient plasma samples using an integrated microfluidic-PANORAMA platform

**Fig. 3:**
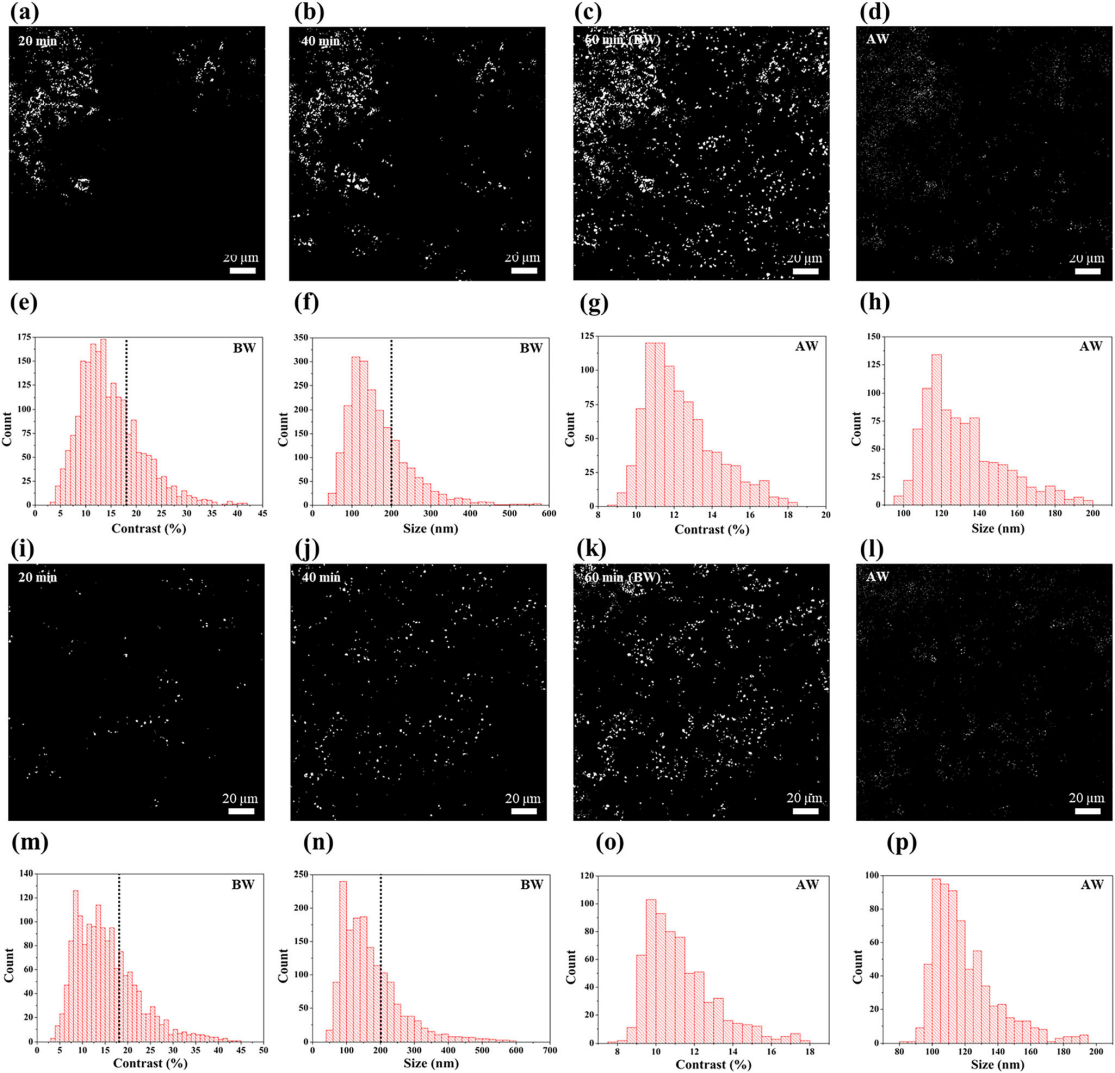
PANORAMA imaging for sEVs detection. Time-lapse PANORAMA images (**a**–**c** for region 1, **i**–**k** for region 2) illustrate the progressive detection of EVs over 60 min (BW), with images captured every 20 min. PANORAMA images (**d** for region 1, **l** for region 2) show sEVs detection (AW). Contrast histograms of EV (BW) (**e** for region 1, **m** for region 2) and diameter (**f** for region 1, **n** for region 2), where the dashed line indicates the cut-off threshold distinguishing sEVs from larger EVs. Contrast histograms of retained sEV (AW) (**g** for region 1, **o** for region 2) and diameter (**h** for region 1, **p** for region 2).

Next, we demonstrated the ability of our device to detect and quantify exosomes directly from the plasma of the second liver cirrhosis patient without any purification. Following MISEV guidelines, we will refer to the detected entities as “sEVs” throughout this section^2^. AGNIS functionalized with biotinylated antibodies CD9, CD63, and CD81 were employed for sEV detection, with the sensing strategy relying on antigen-specific capture to differentiate sEVs from other plasma components. Initially, 20 µL of human plasma was delivered into the microfluidic device at a flow rate of 5 µL/min for 1 min to fill the channel with plasma. Just after the flow stops, images were acquired that serve as the background for processing the PANORAMA images. The “push-pull” incubation and image acquisition protocol described earlier were employed. Steadily increasing sEVs binding to the AGNIS surface have been observed throughout the incubation, as evidenced by time-lapse PANORAMA images (Fig. 3a–c). The number of sEV was 246 at 20 min after plasma introduction and increased to 943 at 40 min and 1553 at 60 min before wash (BW) (Fig. 3a–c). The contrast of the detected EVs at 60 min (BW) was extracted from the PANORAMA images and displayed in Fig. 3e with an average of 12 ± 3%. The contrast was used to estimate the diameter of detected EVs based on the equation in the previous section. The analysis showed that the mean sEV size was 140 ± 33 nm, as shown in Fig. 3f. PANORAMA utilizes optical contrast analysis to achieve single-particle resolution, where intensity changes are directly linked to particle size. This method enables precise differentiation between single sEVs and larger vesicles. The reliability of this approach is supported by size-contrast calibrations and validated through SEM imaging, which provides additional confirmation of individual vesicles captured on the AGNIS surface (Supplementary Note 3 for details). EVs exhibiting less than 18% contrast or a size smaller than 200 nm were categorized as sEVs, while the rest were classified as large EVs. Following the 60-min plasma incubation, referred to as BW, the AGNIS surface was washed by flowing PBS-1X in the channel at a higher flow rate of 5 µL/min for 5 min to remove non-specifically bound EVs. After wash (AW) (Fig. 3d), 893 sEVs were retained on the AGNIS surface, exhibiting a mean contrast of 12.3 ± 1.8% (Fig. 3g). The washing step removed a majority of unbound large sEVs, with the retained sEVs having a mean diameter of 131.4 ± 20.69 nm, as shown in Fig. 3h. These findings highlight the capability of the microfluidic-integrated PANORAMA system to efficiently and effectively capture, count, and characterize sEVs in human plasma without the need for purification and labeling. While mass transfer is a contributing factor in the microfluidic detection of nanoscale particles such as sEVs, it is not the dominant limitation in our platform. The “push–pull” flow strategy, enabled through bidirectional circulation, plays the primary role in enhancing sEV capture. This active flow mechanism significantly improves interactions between sEVs and the antibody-functionalized AGNIS surface, effectively overcoming transport constraints and contributing to the high sensitivity and reproducibility observed across experiments.

To further optimize sEV capture efficiency, different push-pull flow rates were systematically evaluated using the same microfluidic platform and sample volume. Three flow conditions were tested: 0 µL/min (no push-pull), 1.5 µL/min, and 3 µL/min. For both 0 and 1.5 µL/min, a single push-pull cycle lasted 10 min using 20 µL of plasma, while for 3 µL/min, the same sample volume was cycled in 5 min. After incubation, all channels were washed using PBS-1X under the same conditions described earlier to remove unbound or loosely attached particles. At 0 µL/min, 498 sEVs were captured in BW with an average contrast of 10.1 ± 2.3%, and 307 sEVs were retained in AW with a contrast of 8.7 ± 1.5%. At 3 µL/min, 1395 sEVs were detected in BW with an average contrast of 10.8 ± 1.7%, and 722 sEVs were retained in AW with a contrast of 10.4 ± 1.4%. In contrast, the 1.5 µL/min condition resulted in the highest number of sEVs detected in both BW and AW, as detailed earlier in the manuscript. Notably, both 1.5 and 3 µL/min push-pull conditions resulted in substantially higher sEV counts than the 0 µL/min condition, indicating that active flow enhances the likelihood of sEVs encountering and binding to the antibody-functionalized AGNIS surface. However, no further improvement was observed by increasing the flow rate from 1.5 to 3 µL/min; in fact, a slight reduction in sEV count was observed. This outcome suggests that excessively rapid circulation may reduce the effective interaction time between sEVs and the sensing surface, thereby limiting binding efficiency. Based on these results, a flow rate of 1.5 µL/min was selected as the optimal condition to maximize sEV capture while maintaining sufficient interaction time for effective antibody binding (see Supplementary Note 4 for details).

It should be noted that regions 1 and 2, shown in Fig. 1a, of AGNIS were used for detecting sEVs at a flow rate of 1.5 µL/min. To investigate the impact of AGNIS region selection on sEV capture efficiency within the microfluidic channel, we also performed sensing in region 2 (200 µm × 200 µm). PANORAMA experiments in region 2 revealed a gradual increase in sEV detection over time. At 20 min, 185 sEVs were detected, which increased to 624 at 40 min and 1132 at 60 min (Fig. 3i–k). The contrast of the detected sEVs in region 2 was 11.7 ± 3.4% (Fig. 3m), which was used to estimate the mean diameter of the detected sEVs as 126.6 ± 36.5 nm as shown in Fig. 3n. After washing (Fig. 3l), 668 sEVs were retained on the AGNIS surface with the mean contrast of 11.2 ± 1.8% (Fig. 3o) having the mean diameter of 119.8 ± 19.6 nm (Fig. 3p), showing a reduction in particle count compared to region 1, where 893 sEVs were retained. This suggests that exosome capture efficiency is region-dependent within the microfluidic chip, favoring regions near the inlet. In contrast, PANORAMA in PDMS wells showed no significant region-dependent variation in sEV capture efficiency. The serum and plasma samples used in this study were obtained from different individuals. This distinction may explain the observed differences in sizes and contrast values of purified exosomes and sEVs. Personal biological variation is known to affect extracellular vesicle characteristics, including size and concentration, which provides a reasonable explanation for the discrepancy observed between the two sample types.

**Fig. 4:**
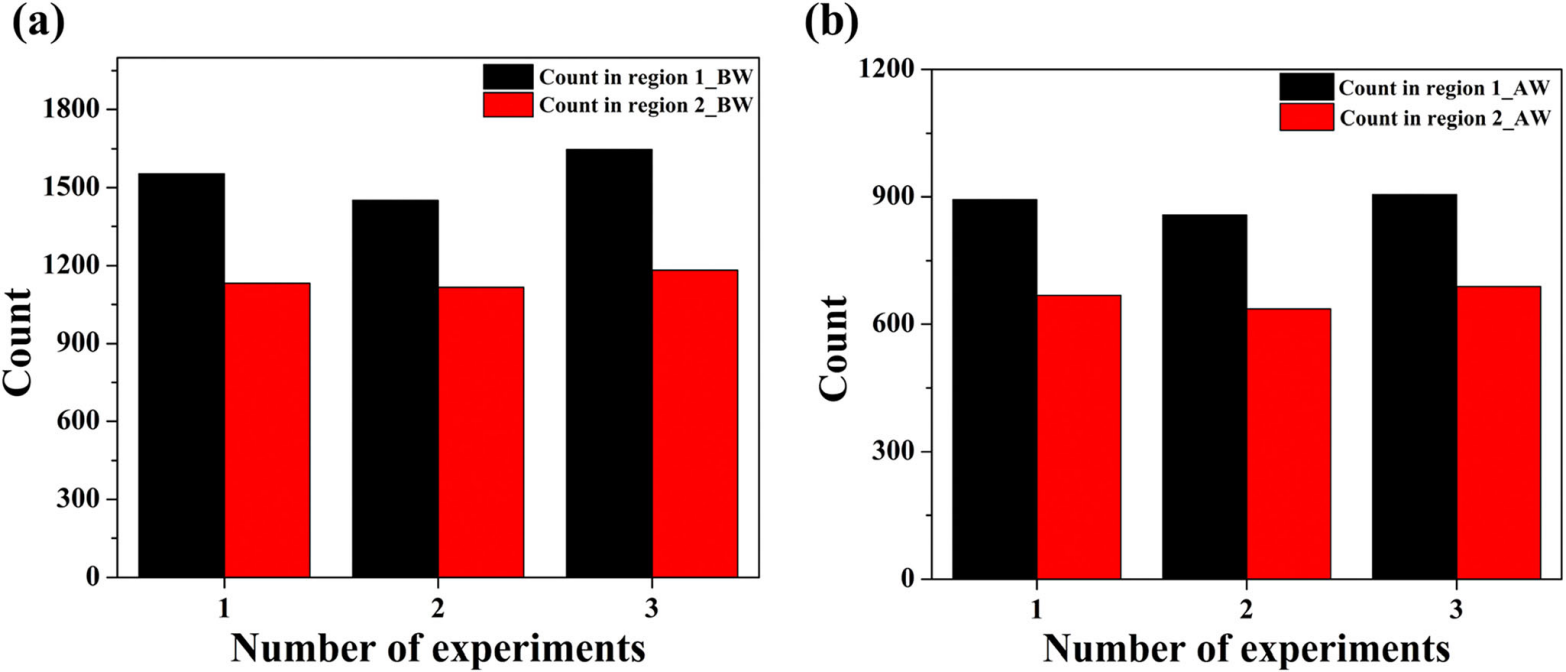
Comparison of BW and AW counts across regions and devices. Bar plots showing **a** BW and **b** AW counts in region 1 and region 2 across three experiments with different devices.

Next, to demonstrate the reproducibility of our integrated microfluidic-PANORAMA platform, we performed experiments using the same liver cirrhosis patient plasma sample on three microfluidic devices. sEV capture was assessed in regions 1 and 2 for both BW and AW images in three independent experiments (see Supplementary Note 5 for details). Figure 4a presents the sEV counts in regions 1 and 2 for BW. In region 1, the sEV counts were 1553, 1451, and 1647, yielding a mean of 1550 ± 98. Region 2 showed counts of 1132, 1116, and 1183, with a mean of 1143 ± 34. Figure 4b shows the AW sEV counts. Region 1 exhibited values of 893, 857, and 905, resulting in a mean of 885 ± 25. Similarly, region 2 showed counts of 668, 636, and 689, with a mean of 664 ± 26. The relatively low standard deviations observed across both regions and conditions underscore the reproducibility and reliability of the experimental approach. These results validate the robustness of the platform and confirm that both regions analyzed yield consistent sEV measurements across independent experiments.

## Discussion

This study demonstrates the purification- and label-free detection, counting, and size characterization of purified exosomes as well as sEVs in human plasma using the PANORAMA technique integrated with an automated microfluidic system. The use of a push-pull flow incubation strategy within the microfluidic device enhanced the interaction between antibodies and the plasma sample, leading to the efficient and effective capture of exosomes/sEVs. With only 20 µL of the sample, purified exosomes as well as sEV in human plasma could be captured, detected, and counted with statistical reproducibility. The platform’s ability to detect sEVs directly from unprocessed plasma, combined with its minimal sample requirement, high sensitivity, and label-free operation, advocates for its potential for fundamental exosome/sEV research and clinical applications. The technical performance demonstrated through consistent results across multiple independently fabricated and functionalized devices underscores the platform’s reproducibility and specificity. However, a key limitation is that all plasma-based experiments were conducted using a single patient-derived sample, which restricts assessment of biological variability across individuals. While the primary goal of this study was to validate the feasibility and robustness of the platform under controlled conditions, future work involving a larger and more diverse set of clinical samples will be critical to fully establish its generalizability and translational potential.

## Methods

### Reagents

Methyl-PEG-thiol [MT(PEG)4] and bovine serum albumin (BSA) were purchased from ThermoFisher Scientific. Thiol-PEG-Biotin was acquired from Nanocs Inc. Polystyrene beads and neutravidin were purchased from Sigma-Aldrich. Biotinylated antibodies CD9, CD63, and CD81 were obtained from BioLegend. Negative (SU8) and positive (AZ1512) photoresists were procured from Microchem and AZ Electronic Materials, respectively. Krayden Dow Sylgard 184 silicone elastomer kit was purchased from ThermoFisher.

### Optical setup of microfluidic-integrated PANORAMA platform

Optical images of the AGNIS were acquired using a standard inverted optical microscope (IX83, Olympus), as illustrated in Fig. 1d. The AGNIS-integrated microfluidic chip, which was connected to a syringe via tubing and powered by a mechanical syringe pump (Chemyx Inc. Model Fusion 720), was placed on the microscope stage. The AGNIS patch within the microfluidic channel was illuminated by a tungsten halogen lamp (U-LH100L3, Olympus) through a 10X condenser lens (IX2-LWUCD, Olympus). To produce the narrowband light, a bandpass filter (FB660-10, Thorlabs) was used. The transmitted light was captured with a 40X objective lens (UPlanSApo 40X/0.95, Olympus) and imaged using a sCMOS camera (Hamamatsu Orca). The exposure time was set to 30 ms per frame with 100 frames averaged, and the imaging area was 200 µm × 200 µm.

### Fabrication of AGNIS-integrated microfluidic device

#### Fabrication of AGNIS

**Fig. 5:**
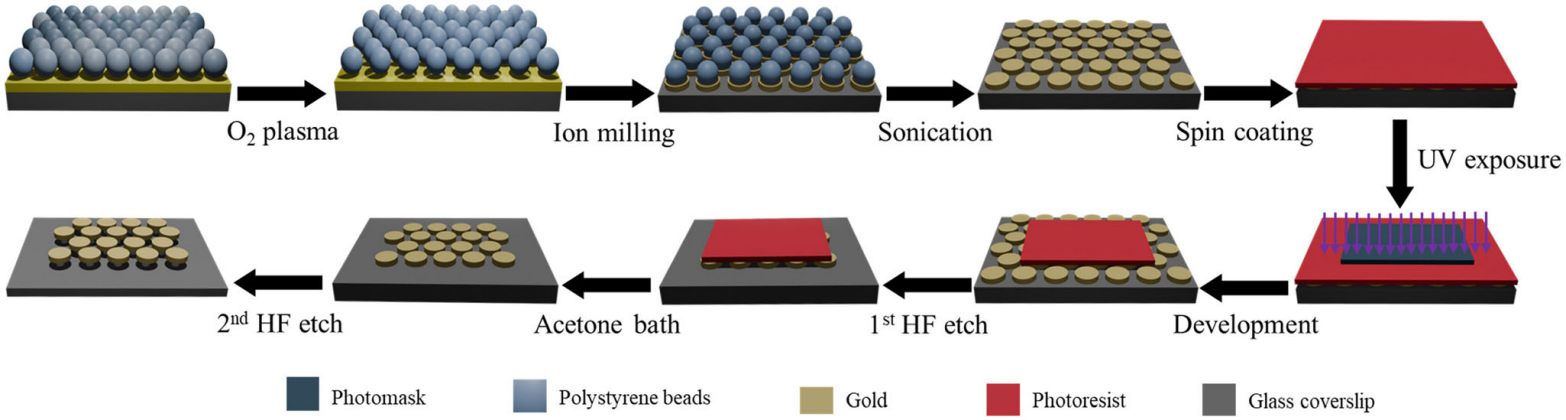
Overview of the fabrication of AGNIS patches. Schematics illustrating the fabrication steps of rectangular AGNIS patches for integration with the microfluidic channel.

AGNIS were fabricated using nanosphere lithography (NSL), as illustrated in Fig. 5. First, a glass coverslip (VWR, No. 2, 24 × 50 mm) was cleaned in acetone, isopropanol, and deionized (DI) water, followed by drying by nitrogen gas. A 2 nm chromium (Cr) adhesion layer and an 80 nm gold (Au) film were then deposited onto the coverslip using DC sputtering at a rate of 3 Å/s. A monolayer of 460 nm polystyrene beads (PSBs) was assembled on the Au surface via the Langmuir–Blodgett technique. The PSB size was reduced to 360 nm using oxygen plasma etching (50 W, 200 V, 30 mTorr), resulting a gap between PSBs that served as the mask for the subsequent argon ion milling to remove Au unmasked by the PSBs. The PSBs were then removed by sonication, yielding an arrayed gold nanodisks (AGN) with a disk diameter of 360 nm over the entire coverslip.

To integrate AGNIS into the microfluidic channel, one is required to bond the AGNIS coverslip with the Polydimethylsiloxane (PDMS) block with the channels. However, the presence of AGN across the entire coverslip surface hinders PDMS-glass plasma bonding. To address this, rectangular AGN patches (1000 µm × 450 µm) were retained on the coverslip, with AGN removed from the remaining areas using photolithography. The AGN surface was uniformly coated with a positive photoresist (AZ1512) and exposed for 3.2 s at 80 mJ/cm² with a lamp power of 25 mW/cm², using a mask with rectangular regions. The photoresist was then developed with AZ 300 MIF, exposing the AGN in the unmasked areas. These exposed regions were etched by immersing the chip in buffered hydrofluoric acid (BHF) until the nanodisks were completely undercut and removed. This process enabled effective bonding of the AGNIS chip to the microfluidic channels. After etching, the remaining photoresist was stripped using acetone, leaving behind precisely patterned rectangular AGN regions. The patterned substrate was further immersed in BHF for 80 s, to create an undercut beneath the nanodisks, forming AGNIS. This undercut structure exposes regions with the highest plasmonic electric-field enhancement, thereby increasing the sensitivity for detecting nanoparticles such as sEVs. The undercut also results in a blue-shifted LSPR peak that enhances compatibility with visible and near-infrared light sources and detectors, and better diffraction-limited resolution. Figure 1b shows a representative image of a section of the AGNIS.

#### Fabrication of microfluidic channel and integration with AGNIS

**Fig. 6:**
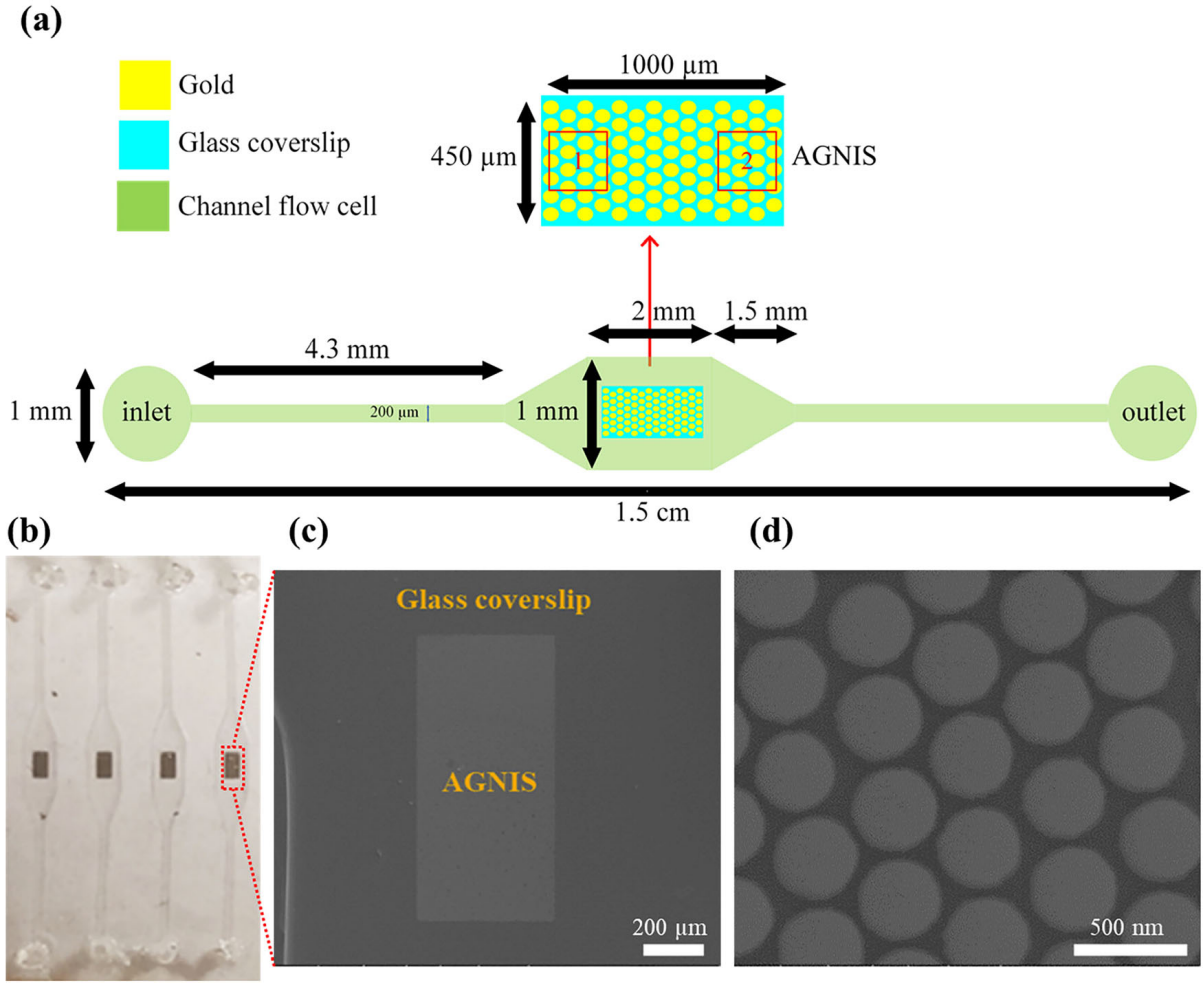
Details of the microfluidic channel. **a** Microfluidic channel design. **b** Optical image of the AGNIS-integrated microfluidic device. **c** SEM image of a single AGNIS patch within the microfluidic channel. **d** SEM image (top view) of densely packed AGNIS from a small area in (**c**).

Soft lithography was used to fabricate microfluidic devices. A SU-8 mold was created on a silicon wafer using standard photolithography techniques (see Supplementary Note 6 for fabrication details). The design features four identical channels, each with an inlet, outlet, and middle section, as illustrated in Fig. 6a. The inlet and outlet sections are identical with a width of 200 µm and length of 4.3 mm. The middle segment is 2 mm in length and 1 mm in width. The middle section is wider than the inlet/outlet to accommodate the AGNIS (1000 µm × 450 µm) and facilitate manual alignment during bonding. The thickness of the channel was set to 100 µm.

Polydimethylsiloxane (PDMS) was prepared by mixing silicon elastomer and curing agent in a 10:1 ratio, then poured onto the SU-8 mold and cured at 70 °C for 2 h. After curing, the PDMS structure was peeled from the mold, and inlet and outlet channels were punched with a 1 mm biopsy puncher, followed by cleaning with acetone, isopropanol, and deionized water. The microfluidic channels were bonded to the AGNIS chip using oxygen plasma treatment to ensure a strong PDMS-glass bond. This bonding strategy deliberately incorporates small gaps between the AGNIS and the channel walls to ensure strong adhesion and minimize the risk of leakage during operation. Although extending AGNIS coverage might enhance capture efficiency, this approach effectively balances operational functionality with structural stability. Figure 6b shows the AGNIS-integrated microfluidic device with four channels and rectangular AGNIS patches in the middle section. The SEM images in Fig. 6c, d show, respectively, a low-magnification view of the full rectangular AGNIS chip and a high-magnification view highlighting the densely packed nanodisks within the microfluidic channel.

#### Surface functionalization of AGNIS within the microfluidic channel

To functionalize the AGNIS surface within the microfluidic channel, a serial incubation process was employed. First, a 1:3 mixture of long-chain active biotin-PEG-thiol (2 mM, MW:1 kDa) and short inactive methyl-PEG-thiol (2 mM, MW: 0.2 kDa) was introduced into the channel with a flow rate of 5 µL/min using a syringe pump (Chemyx Inc., Model Fusion 720). The microfluidic chip was then incubated for approximately 16 h, allowing the thiol groups to bind strongly with the Au nanodisks. After incubation, the channels were washed with phosphate-buffered saline (PBS-1X) with a flow rate of 20 µL/min to remove unbound PEG-thiol molecules. Next, neutravidin with a flow rate of 5 µL/min was introduced as a linker molecule and incubated for 2 h, followed by thorough washing. Finally, a mixture of exosome-specific biotinylated antibodies (CD9, CD63, and CD81 with a concentration of 50 µg/mL) diluted with 2.5% BSA is flown through the channel with a flow rate of 5 µL/min and incubated for an additional 2 h. It should be noted that all the incubations were done at 4 °C. Figure 1c illustrates the AGNIS surface after functionalization. The AGNIS-integrated microfluidic chip was then used for sEVs detection via antibody-antigen-specific binding.

#### Image processing

In PANORAMA imaging of purified exosomes and plasma-derived EVs, image processing is key for accurate, label-free detection. First, a background image (before adding the sample) and a sample image (after exosome binding) are acquired and carefully aligned using ImageJ to ensure pixel-level precision. A ratiometric image, or intensity ratio (IR) image, is then created by dividing the sample image by the background image, where IR values above 1 indicate the presence of particles due to increased light transmission from local refractive index changes. To distinguish true exosome signals from the background, a statistical threshold is applied, calculated as IR = 1 + 3*σ* + 0.005, where σ is the standard deviation of background IR values and 0.005 (or 0.5%) is an added safety margin^30^. Pixels exceeding this threshold are considered detected exosomes or EVs. For each detected particle, contrast is calculated as (IR − 1) × 100%. This streamlined image processing approach ensures high sensitivity and accuracy for analyzing both purified and complex biological samples.

#### Procedure for plasma extraction

All participants provided consent under the institutional review board (IRB)-approved biomarker protocols at MD Anderson Cancer Center. Patients also consented to IRB-approved prospective biomarker or therapeutic protocols based on their disease site. Blood samples were collected via venipuncture before the initiation of treatment (radiation, surgery, or chemotherapy), and the extracted plasma was aliquoted and stored at −80 °C until use. The analysis of these samples for sEV research was conducted under protocol 2021-0368, which permits the use of de-identified samples from consented patients enrolled in IRB-approved studies. The approved IRB ID is 2021- 0368_CR001, with registration ID IRB 2 IRB00002203.

## Supplementary information

Supplementary Information

## Data Availability

All source data supporting this study's findings are available from the corresponding author upon reasonable request.
