# Supplementary material for: Microfluidic nano-plasmonic imaging platform for purification- and label-free single small extracellular vesicle characterization
Source: NPJ Biosens. 2025 Jul 30;2(1):26. doi: 10.1038/s44328-025-00047-w (PMC12310528; doi:10.1038/s44328-025-00047-w)
Supplement: Supplementary file 1 — Supplementary Information [file 44328_2025_47_MOESM1_ESM.docx]

**Microfluidic Nano-Plasmonic Imaging Platform for Purification- and Label-Free Single Small Extracellular Vesicle Counting**

Omid Mohsen Daraei^1^, Avinash Kumar Singh^1^, Saswat Mohapatra^1^, Mohammad Sadman Mallick^1^, Abhay Kotnala^1^, and Wei-Chuan Shih^1,2,3,4,*^

^1^Department of Electrical and Computer Engineering, University of Houston, 4800 Calhoun Road, Houston, Texas 77204, United States of America

^2^Department of Biomedical Engineering, University of Houston, 4800 Calhoun Road, Houston, Texas 77204, United States of America

^3^Department of Chemistry, University of Houston, 4800 Calhoun Road, Houston, Texas 77204, United States of America

^4^Program of Materials Science and Engineering, University of Houston, 4800 Calhoun Road, Houston, Texas 77204, United States of America

* Corresponding author and email: wshih@central.uh.edu

**Supplementary Note 1:**

The optical configuration for acquiring LSPR spectra of AGNIS was constructed using an inverted Olympus microscope (IX71). The AGNIS sample was illuminated by a 100W white halogen lamp through a 0.55 NA condenser. The transmitted light was captured by a 40X/0.75 numerical aperture (NA) objective lens (UplanFLN40X) and directed to a spectrometer (Princeton Instruments, Acton 2300) equipped with a thermoelectrically cooled (-70°C) CCD camera (Princeton Instruments, PIXIS 400) via a 4-f system. A schematic of the optical setup is presented in Supplementary Figure 1a. AGNIS exhibited LSPR peak wavelengths of 659 nm in air and 739 nm in water, corresponding to a sensitivity of 242.42 nm/RIU (Supplementary Figure 1b).

| **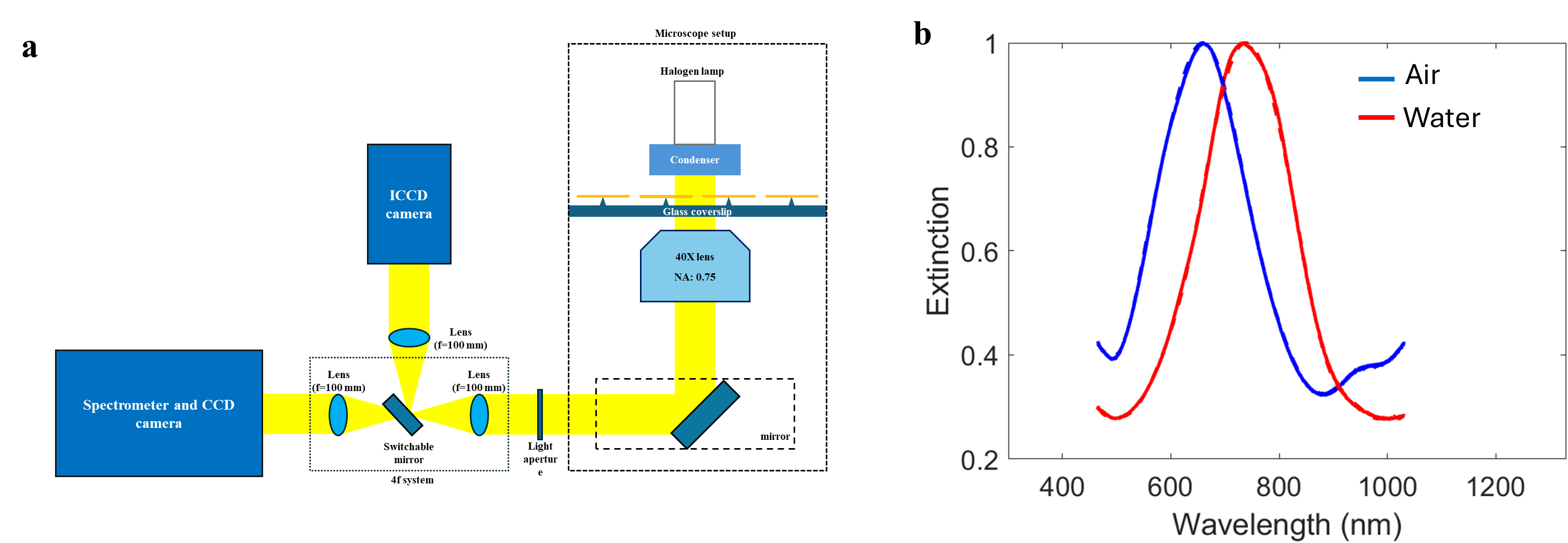**  *Supplementary Figure 1: a) Schematic of LSPR imaging system setup, b) LSPR spectra of AGNIS in air and water medium.* |
| --- |

**Supplementary Note 2:**

Exosome detection was carried out at four different concentrations: 7.2×10⁴ exosomes/μl, 7.2×10⁵ exosomes/μl, 7.2×10⁶ exosomes/μl, and 7.2×10⁷ exosomes/μl. The results of these experiments are detailed below, with references to the corresponding figures for clarity.

At a concentration of 7.2×10⁴ exosomes/μl, the numbers of detected exosomes in experiments 1, 2, and 3 were 83, 79, and 94, respectively (Supplementary Figure 2a-c). The corresponding exosome contrasts for these experiments were measured as 10.2±1.7 %, 8.3±0.8 %, and 9.7±2.2 %, respectively (Supplementary Figure 2d-f).

At a concentration of 7.2×10⁵ exosomes/μl, the numbers of detected exosomes in experiments 1, 2, and 3 were 537, 515, and 498, respectively (Supplementary Figure 2g-i). The corresponding exosome contrasts for these experiments were measured as 9.1±1.5 %, 8.6±1.7 %, and 9.5±1.1 %, respectively (Supplementary Figure 2j-l).

At a concentration of 7.2×10⁶ exosomes/μl, the numbers of detected exosomes in experiments 1, 2, and 3 were 968, 1031, and 887, respectively (Supplementary Figure 3a-c). The corresponding exosome contrasts for these experiments were measured as 8.7±1.5 %, 9.3±1.9 %, and 9.2±1.5 %, respectively (Supplementary Figure 3d-f).

At a concentration of 7.2×10⁷ exosomes/μl, the numbers of detected exosomes in experiments 1, 2, and 3 were 2563, 2458, and 2676, respectively (Supplementary Figure 3g-i). The corresponding exosome contrasts for these experiments were measured as 8.7±1.6 %, 9.0±2.4 %, and 10.8±2.0 %, respectively (Supplementary Figure 3j-l). These results demonstrate the relationship between exosome concentration and detection efficiency, with higher concentrations yielding larger counts of detected exosomes. The measured exosome contrasts remain within a relatively narrow range across different concentrations and experimental repetitions, indicating the reproducibility of the detection process.

| **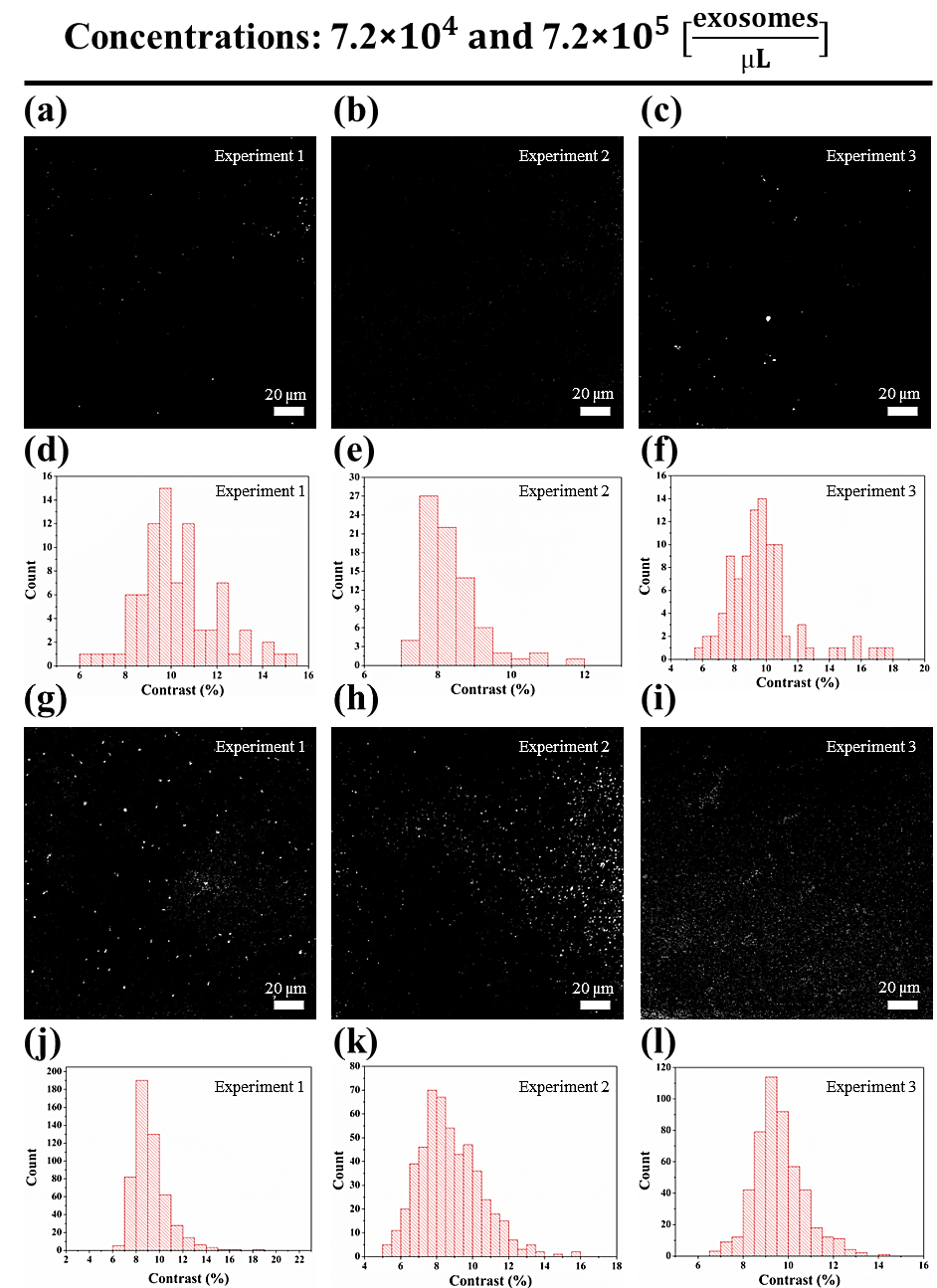**  ***Supplementary Figure 2****: (a-c) PANORAMA images illustrating purified exosome detection from experiments 1 to 3 at concentrations of 7.2×10^4^ exosomes/µl. (d-f) Contrast histograms of the detected exosomes corresponding to (a-c). (g-i) PANORAMA images illustrating purified exosome detection from experiments 1 to 3 at a concentration of 7.2×10^5^ exosomes/µl. (j-l) Contrast histograms of the detected exosomes corresponding to (g-i).* |
| --- |

| ***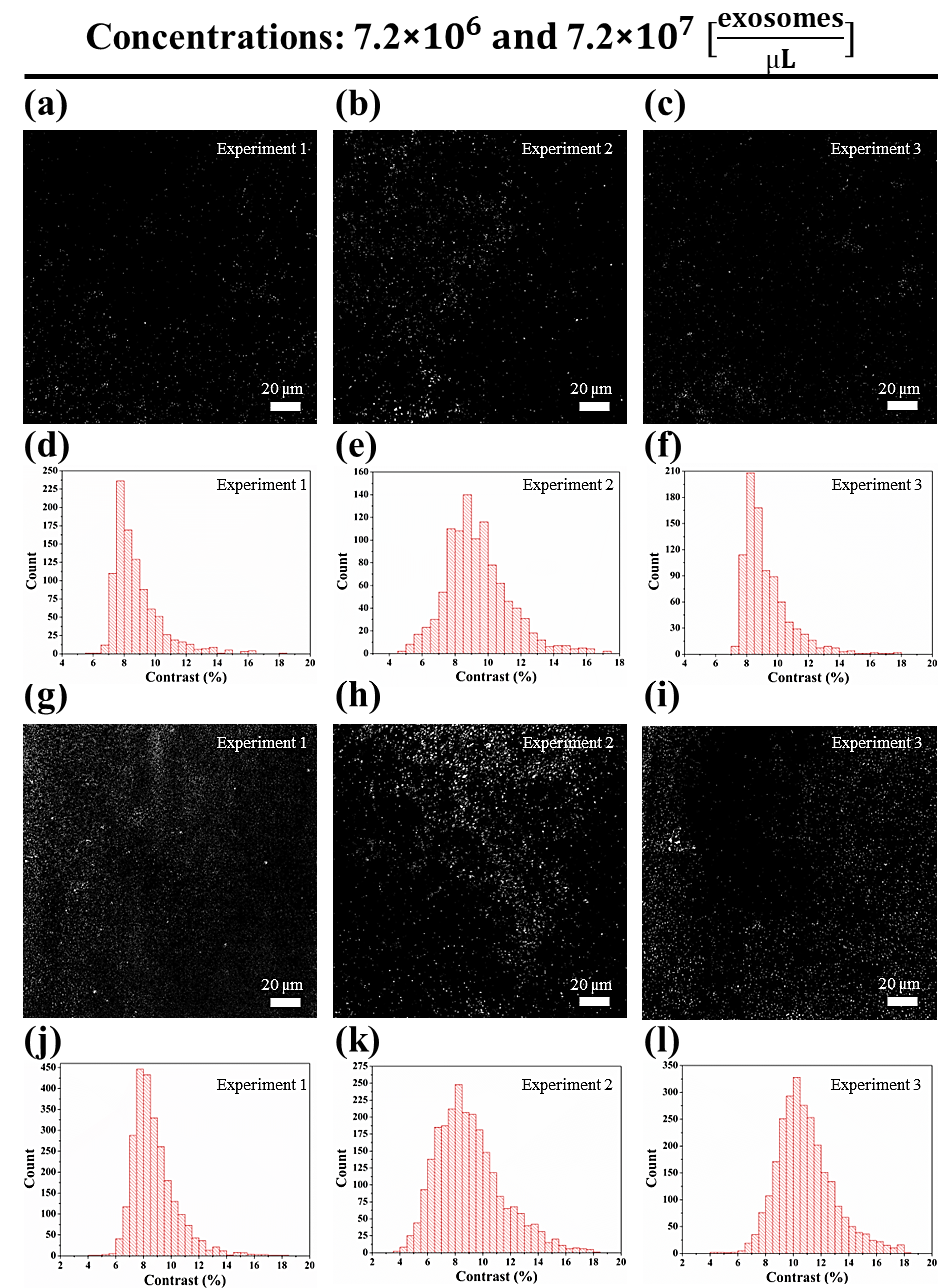***  ***Supplementary Figure 3****: (a-c) PANORAMA images illustrating purified exosome detection from experiments 1 to 3 at concentrations of 7.2×10⁶ exosomes/µl. (d-f) Contrast histograms of the detected exosomes corresponding to (a-c). (g-i) PANORAMA images illustrating purified exosome detection from experiments 1 to 3 at a concentration of 7.2×10⁷ exosomes/µl. (j-l) Contrast histograms of the detected exosomes corresponding to (g-i).* |
| --- |

**Supplementary Note 3:**

The SEM images provide further direct evidence of individual sEV captured on AGNIS (Supplementary Figure 4a,b).

| 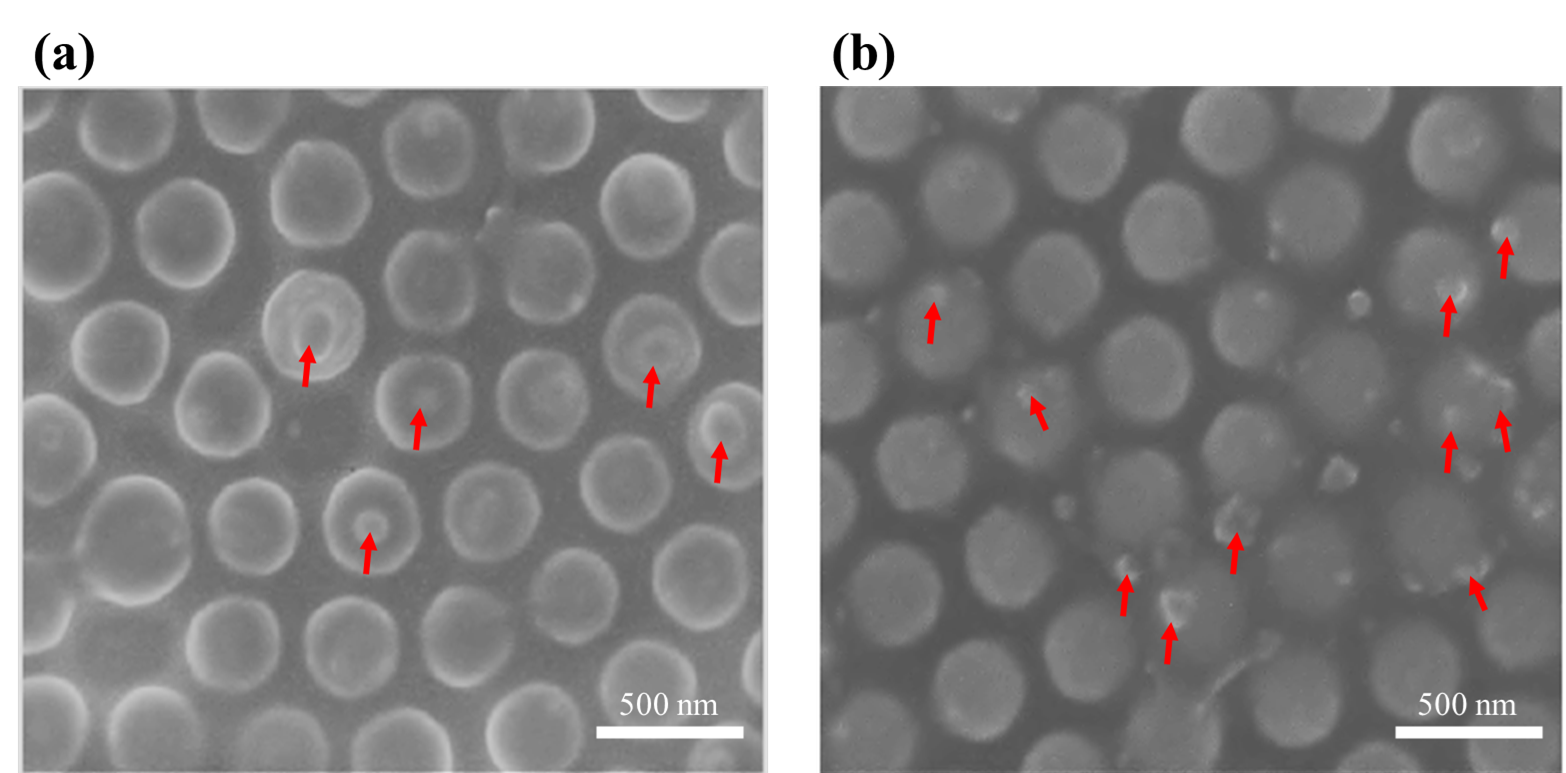  *Supplementary Figure 4: (a,b) SEM images displaying sEVs on AGNIS, illustrated by red arrows.* |
| --- |

**Supplementary Note 4:**

To evaluate the influence of different push-pull flow rates on sEV capture efficiency, three flow conditions—3 µL/min, 1.5 µL/min, and 0 µL/min (no push-pull)—were tested using the same plasma sample and a fixed plasma volume of 20 µL. All experiments were conducted for the same total incubation time of 60 minutes. In the 3 µL/min condition, the plasma sample was actively recirculated through the microfluidic channel for the entire 60-minute duration. Under this condition, 1395 sEVs were captured in BW (Supplementary Figure 5a) with an average contrast of 10.8±1.7 % and a threshold of 3.9 % (Supplementary Figure 5c), while 722 sEVs were retained in AW (Supplementary Figure 5b) with an average contrast of 10.4±1.4 % and a threshold of 3.8 % (Supplementary Figure 5d). In the 0 µL/min (no push-pull) condition, the plasma sample remained stationary within the microfluidic channel for the full 60 minutes. This condition resulted in 498 sEVs captured in BW (Supplementary Figure 5e) with an average contrast of 10.1±2.3 % and a threshold of 3.7 % (Supplementary Figure 5g), while 307 sEVs were detected in AW (Supplementary Figure 5f) with an average contrast of 8.7±1.5 % and a threshold of 3.7 % (Supplementary Figure 5h). The 1.5 µL/min condition also involved continuous recirculation for 60 minutes. This flow rate yielded the highest number of sEVs in both BW and AW, as detailed in the main manuscript. These findings demonstrate that active push-pull flow enhances sEV capture compared to static conditions. Both 1.5 and 3 µL/min significantly outperformed the no-flow setup. However, increasing the flow rate from 1.5 to 3 µL/min did not result in further improvement and slightly reduced sEV counts. Based on these results, 1.5 µL/min was selected as the optimal flow rate, balancing active circulation with effective surface interaction over the 60-minute incubation period.

| 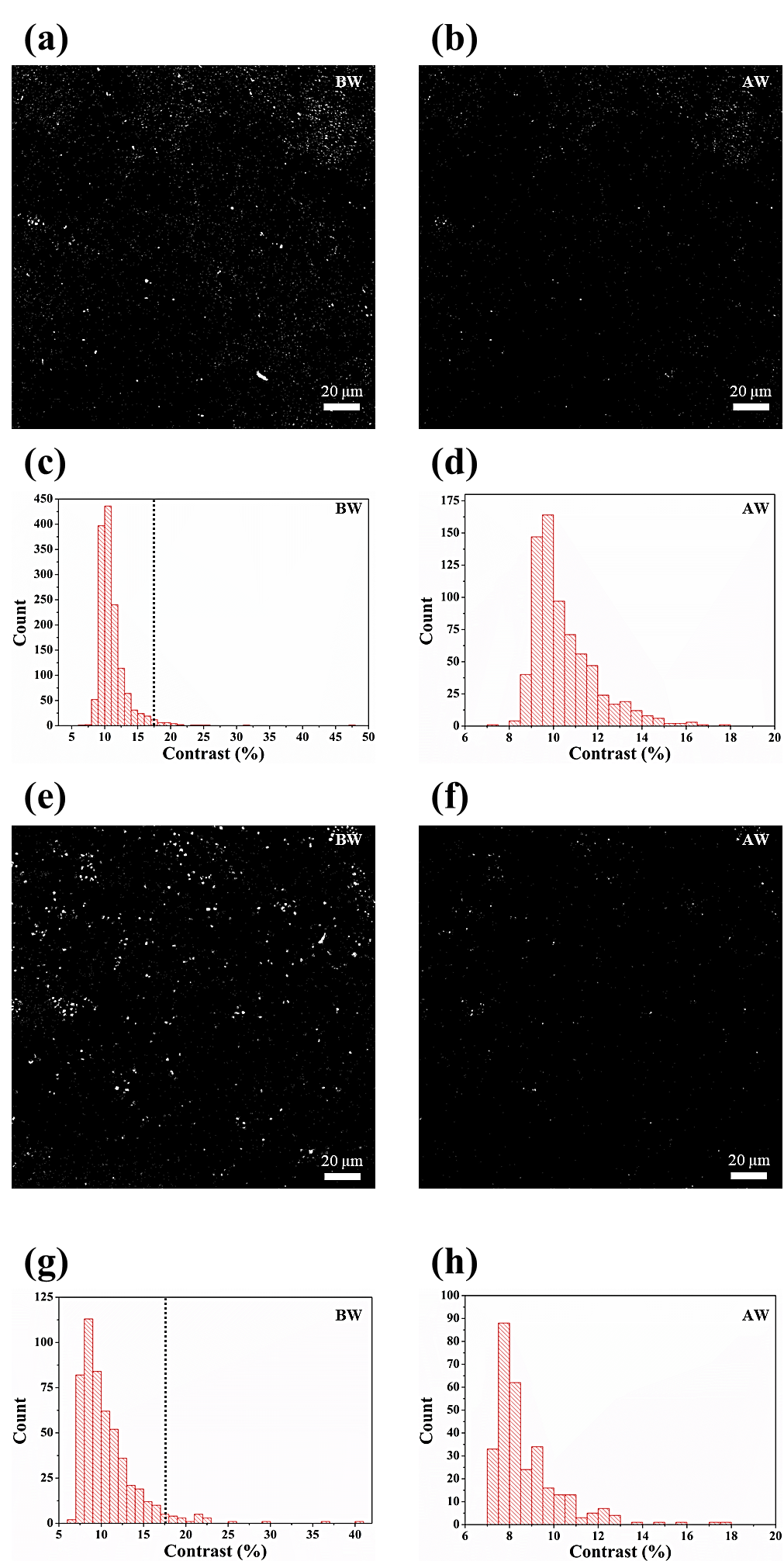 |
| --- |

*Supplementary Figure 5: (a,e) PANORAMA images showing EV detection at 60 minutes (BW) for the flow rates of 3 µL/min and 0 µL/min (no push-pull), respectively. (b,f) PANORAMA images illustrating sEV detection (AW) at the same flow rates. (c,g) Contrast histograms of EVs (BW), corresponding to (a,e), where the dashed line indicates the cut-off threshold distinguishing sEVs from larger EVs. (d,h) Contrast histograms of retained sEVs (AW), corresponding to (b,f). Dashed lines indicate the contrast threshold separating sEVs from larger EVs.*

**Supplementary Note 5:**

The PANORAMA experiments demonstrated a significant variation in sEV capture efficiency between the two AGNIS regions within the microfluidic channel. To ensure the reproducibility of sEV counts in region 1 and region 2, two additional experiments were conducted in both regions, revealing consistent capture trends and validating the reliability of the system.

In experiment 2, region 1 for BW was analyzed with a threshold of 3.7 %, resulting in a count of 1451 sEVs (Supplementary Figure 6a) and a contrast of 11.4±2 % (Supplementary Figure 6e), equivalent to a mean size distribution of 122.3±22.1 nm (Supplementary Figure 6f), with a retention rate of 59 %. For AW in region 1, a threshold of 3.6 % resulted in a count of 857 sEVs (Supplementary Figure 6b) and a contrast of 10.2±1.9 % (Supplementary Figure 6i), equivalent to a mean size distribution of 109.5±20.7 nm (Supplementary Figure 6j). In region 2, BW with a threshold of 3.8 % recorded a count of 1116 sEVs (Supplementary Figure 6c) and a contrast of 11.6±1.9 % (Supplementary Figure 6g), equivalent to a mean size distribution of 123.5±21.3 nm (Supplementary Figure 6h), with a retention rate of 57 %. For AW in region 2, a threshold of 3.5 % yielded a count of 636 sEVs (Supplementary Figure 6d) with a contrast of 10.9±1.7 % (Supplementary Figure 6k), equivalent to a mean size distribution of 116.1±18 nm (Supplementary Figure 6l).

In experiment 3, region 1 BW had a threshold of 3.8 %, resulting in an sEV count of 1647 (Supplementary Figure 7a) and a contrast of 12.4±2.3 % (Supplementary Figure 7e), equivalent to a mean size distribution of 132.4±25.7 nm (Supplementary Figure 7f). The retention rate for this region was recorded at 54.9 %. For AW in region 1, a threshold of 3.5 % produced an sEV count of 905 (Supplementary Figure 7b) and a contrast of 10.2±1.7 % (Supplementary Figure 7i), equivalent to a mean size distribution of 108.8±18.4 nm (Supplementary Figure 7j). In region 2, BW with a threshold of 3.9 % recorded a count of 1183 sEVs (Supplementary Figure 7c) and a contrast of 11.6±2 % (Supplementary Figure 7g), equivalent to a mean size distribution of 123.3±21.5 nm (Supplementary Figure 7h), with a retention rate of 58.24 %. Finally, AW in region 2, with a threshold of 3.8 %, resulted in a count of 689 sEVs (Supplementary Figure 7d) and a contrast of 10.3±1.5 % (Supplementary Figure 7k), equivalent to a mean size distribution of 109.9 ± 16.5 nm (Supplementary Figure 7l). The data highlights the differences in performance between the two methods and the variability across regions. This suggests higher exosome capture efficiency in the AGNIS region near the inlet, likely due to the sample's initial interaction with AGNIS in region 1, where all sEVs in the plasma are available for binding. As the sample flows forward, the availability of sEVs decreases, reducing capture efficiency near region 2. Therefore, region 1 provides optimal conditions for maximizing capture efficiency, making it a preferred choice for sEV detection within the microfluidic channel.

| **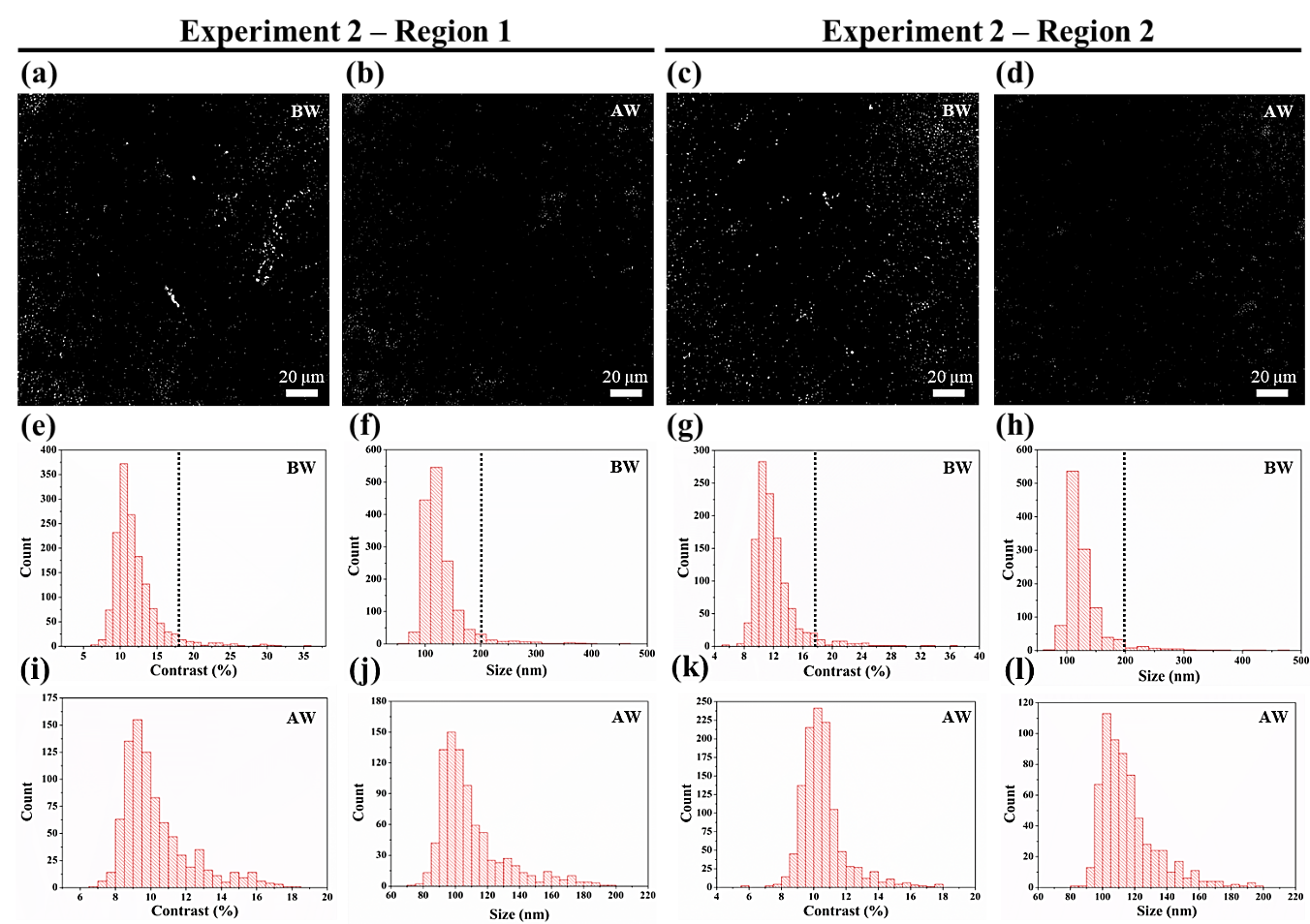**  ***Supplementary Figure 6****: (a,c) PANORAMA images showing EV detection at 60 minutes (BW) in regions 1 and 2 for experiment 2. (b,d) PANORAMA images illustrating sEV detection (AW) in regions 1 and 2 for the same experiment. Contrast histograms of EV (BW) (e for region 1, g for region 2) and diameter (f for region 1, h for region 2, where the dashed line indicates the cut-off threshold distinguishing sEVs from larger EVs. Contrast histograms of retained sEV (AW) (i for region 1, k for region 2) and diameter (j for region 1, l for region 2).* |
| --- |

| 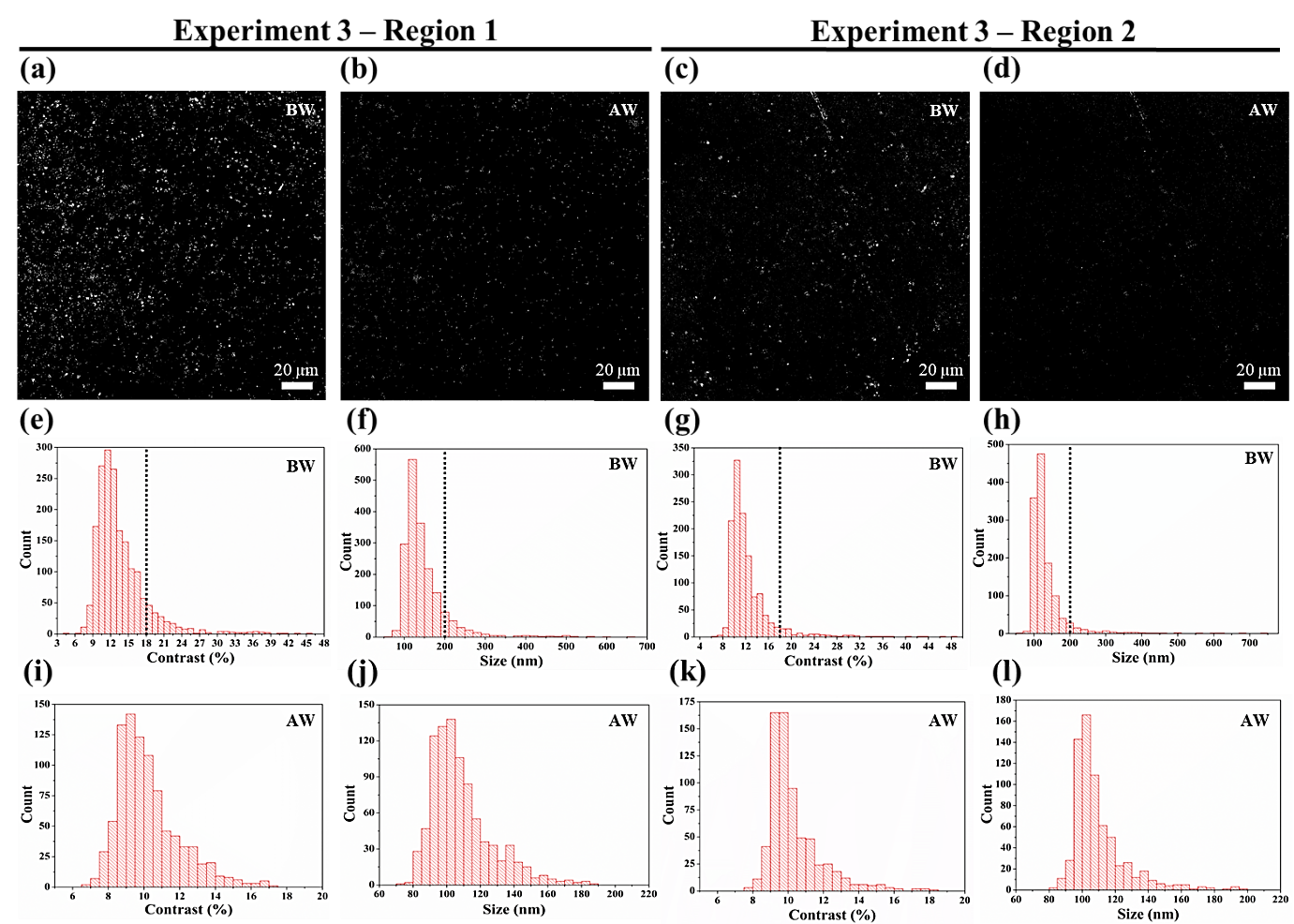  ***Supplementary Figure 7: (a,c) PANORAMA images showing EV detection at 60 minutes (BW) in regions 1 and 2 for experiment 3. (b,d) PANORAMA images illustrating sEV detection (AW) in regions 1 and 2 for the same experiment.*** *Contrast histograms of EV (BW) (e for region 1, g for region 2) and diameter (f for region 1, h for region 2, where the dashed line indicates the cut-off threshold distinguishing sEVs from larger EVs. Contrast histograms of retained sEV (AW) (i for region 1, k for region 2) and diameter (j for region 1, l for region 2).* |
| --- |

**Supplementary Note 6:**

Fabricating polydimethylsiloxane (PDMS) was used for producing our microfluidic device. The process involves casting PDMS onto a patterned SU-8 mold on a silicon wafer. The following steps outline the fabrication of PDMS channel flow cells (Supplementary Figure 8):

1. Clean a 4-inch silicon wafer with acetone, deionized water (DI), and isopropanol (IPA) to remove contaminants.
2. Spin-coat SU-8 photoresist on the wafer at 2000 rpm for 30 seconds. Perform a soft bake at 65 °C for 5 minutes, followed by 95 °C for 20 minutes.
3. Expose the SU-8 to UV light at 250 mJ/cm² for 10 seconds (lamp power 25 mW/cm²), followed by post-exposure bake at 65 °C for 5 minutes, then 95 °C for 10 minutes.
4. Develop the SU-8 pattern in developer for 10 minutes, rinse with DI water, dry with nitrogen gas, and perform a hard bake at 150 °C for 10 minutes.
5. Mix PDMS (10:1 ratio of base to curing agent) and pour over the SU-8 mold. Cure at 70 °C for 2 hours.
6. Peel the cured PDMS, treat with oxygen plasma at 50 W for 30 seconds to enhance hydrophilicity.
7. Transfer the PDMS channel flow cells onto arrayed gold nanodisk substrates (AGNIS) for further use.

| **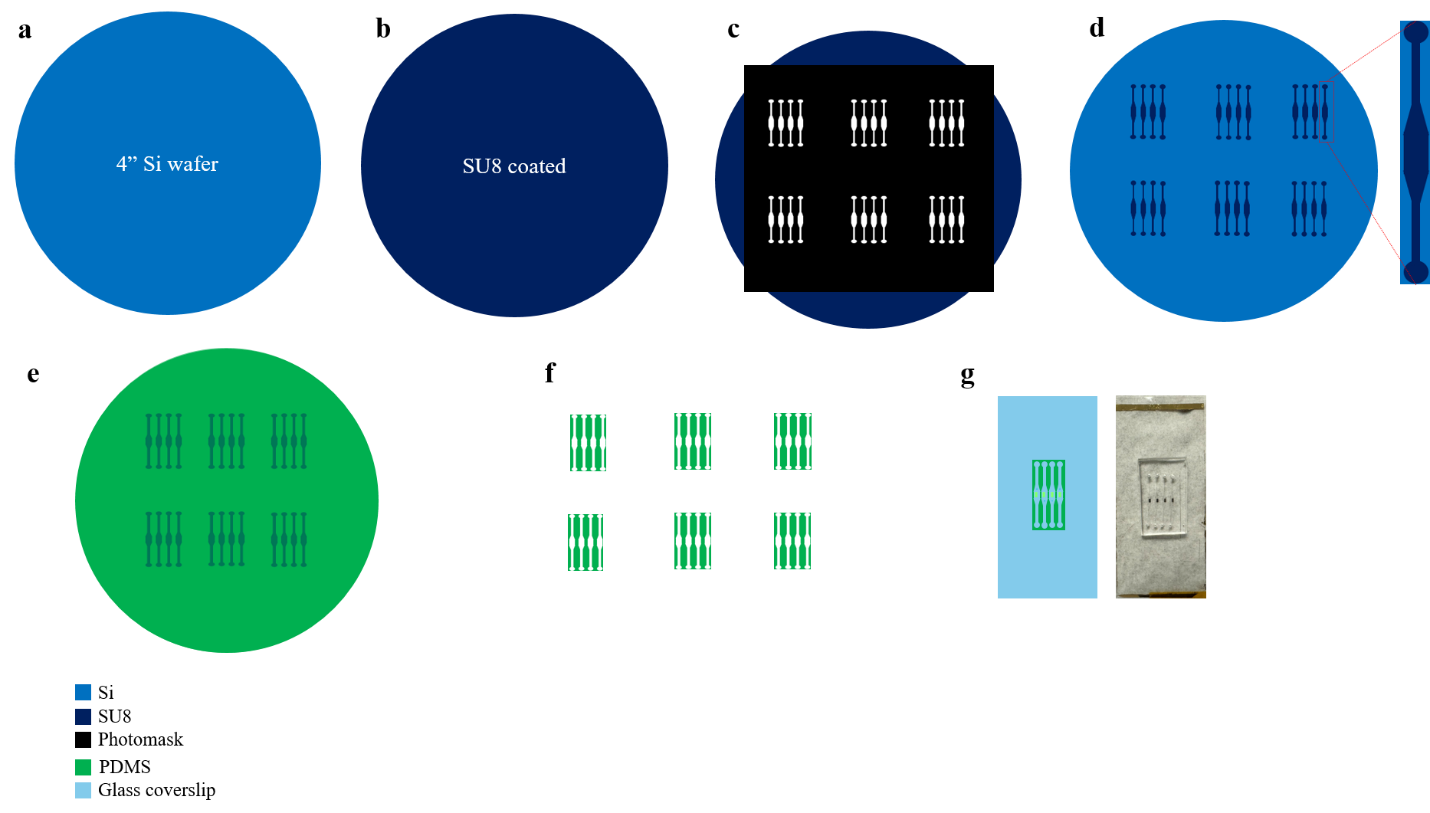**  *Supplementary Figure 8: Fabrication steps of PDMS flow cell.* |
| --- |

**Supplementary Note 7:**

Sensitivity: PANORAMA demonstrated the ability to detect and quantify sEVs at concentrations as low as 1×10⁴ sEVs/mL for sEV detection, corresponding to a limit of detection (LOD) of 16.7 aM ^1^​, which is much better than enzyme-linked immunosorbent assays (ELISA) (1×10⁸ sEVs/mL) ​^2,3^ and nanoparticle tracking analysis (NTA) (1×10⁸–8×10⁸ sEVs/mL)​ ^4^. This enhancement in sensitivity can be attributed to the AGNIS structure, which significantly enhances the electric field and, consequently, the detection signal. Although our concentration detection limit is comparable to the nPLEX platform (~4.04×10^5^ sEVs/mL) ^3^​, our single-particle detection sensitivity is far better than that of nPLEX, which does not provide single-particle analysis. Specificity: Our platform uses a capture-assisted isolation method with multiple antibodies (CD9, CD63, and CD81) simultaneously. This strategy increases the specificity by ensuring that only vesicles that express all of these markers are captured and analyzed. In contrast, in a standard sandwich ELISA, only two antibodies (capture and detection) are typically used. Response Time: Our PANORAMA system completes analysis within 60 minutes, whereas ELISA typically requires 2 to 6 hours due to the blocking, incubation, and washing steps​.

Specificity: Our platform uses a capture-assisted isolation method with multiple antibodies (CD9, CD63, and CD81) simultaneously. This strategy increases the specificity by ensuring that only vesicles that express at least one all of these markers are captured and analyzed.

Supplementary Table 1

| Technique | LOD | Response Time | Reference |
| --- | --- | --- | --- |
| ELISA | 1×10⁸ sEVs/mL | 2–6 hours | ​​^2,3^ |
| NTA | ~1×10⁸ sEVs/mL | 10–30 minutes | ^4^ |
| nPLEX | ~4.04×10^5^ sEVs/mL | 1–2 hours | ^3^​ |
| PANORAMA | 1×10⁴ sEVs/mL | 60 minutes | ^1^ |

**References:**

1. Ohannesian, N. et al. Plasmonic nano-aperture label-free imaging of single small extracellular vesicles for cancer detection. *Communications Medicine* **4**, 100 (2024).
2. Leonardi, A. A. et al. A novel silicon platform for selective isolation, quantification, and molecular analysis of small extracellular vesicles. *Int J Nanomedicine* **16**, 5153–5165 (2021).
3. Im, H. et al. Label-free detection and molecular profiling of exosomes with a nano-plasmonic sensor. *Nat Biotechnol* **32**, 490–495 (2014).
4. Dragovic, R. A. et al. Sizing and phenotyping of cellular vesicles using nanoparticle tracking analysis. *Nanomedicine* **7**, 780–788 (2011).
